# The Use of Computer Records: A Tool to Increase Productivity in Dairy Herds

**DOI:** 10.3390/ani10010111

**Published:** 2020-01-10

**Authors:** Zazil Sánchez, Carlos Salvador Galina, Bernardo Vargas, Juan José Romero, Sandra Estrada

**Affiliations:** 1 Department of Reproduction, Faculty of Veterinary Medicine and Zootechnics, National Autonomous University of Mexico, Mexico City 04510, Mexico; zazilsnchz@gmail.com (Z.S.); cgalina@unam.mx (C.S.G.); 2 Regional Postgraduate in Tropical Veterinary Sciences, Veterinary Medicine School, National University, Heredia 40101, Costa Rica; 3 Research Program in Population Medicine, Veterinary Medicine School, National University, Heredia 40101, Costa Rica; juan.romero.zuniga@una.cr (J.J.R.); sandra.estrada.konig@una.cr (S.E.)

**Keywords:** information systems, dairy records, milk, production

## Abstract

**Simple Summary:**

The objective of this paper was to evaluate the use of records in cattle production systems in order to determine and quantify the possible benefits on farm productivity and the economic benefit associated with their implementation. The results suggest that use of records contributes to cattle management improvement, opening a window to implement information and communication technologies in order to make management decisions based on certified information, which in turn will improve the efficiency of cattle production systems in developing countries.

**Abstract:**

The level of adoption of herd management information system veterinary automated management and production control program (VAMPP) Bovine and its impact on productivity of 912 specialized dairy systems with at least 5 years of information recorded was studied. Herds were classified as low (*n* = 389), medium (*n* = 343), or high (*n* = 180) adoption level on the basis of extent and consistency of record keeping for variables related to production, reproduction, and health. For each herd, within-year averages were obtained for six performance traits: age at first calving (AFC), days open (DO), daily milk yield (DMY), productive life (PL), incidence of clinical mastitis (MAST), and incidence of lameness (LAM). These variables were investigated with a generalized linear mixed model that included the fixed effects of the adoption level, follow-up year, and their interaction, adjusted by the fixed effects of herd size, agroecological zone, calendar period, breed group, and the random effects of variation between/within herds. A significant effect of the adoption level over all the variables was observed, except DMY and PL. The follow-up year was significant for all the variables except LAM. There were marked reductions for AFC and DO in the first 4 years of follow-up. AFC was higher and DO shorter in the low compared to medium and high level of adoption herds (*p* < 0.001). DMY showed a significant increasing trend (*p* < 0.001), regardless of the adoption level. There was higher MAST and LAM incidence in the higher adoption level (*p* < 0.05). The economic benefit was estimated through a stochastic simulation model using an approach based in partial budget analysis. For a herd with a medium level of adoption, the change in gross margin (GM_MIS_ $USD) and marginal return rate (MRR_MIS_ %) for the first 5 years of use of the system was estimated. Under these conditions, there was a GM_MIS_ of $6890 and MRR_MIS_ of 163%. Variation of ±10% in DMY and DO caused changes in the GM_MIS_ of ±$1000 and ±$110, respectively, and in MRR_MIS_ ±24% and ±4%, respectively. The trends suggest a positive influence of VAMPP in productive and reproductive traits during the first years of implementation, with less benefit for the low adoption levels.

## 1. Introduction

To have a productive and efficient dairy production system, the producer should be able to evaluate the impact of each event and measure taken in the farm [1], or if not all, at least of the ones with the highest priority or impact on farm performance. Record-keeping is the most efficient tool to know the dynamics of production and productivity, as well as the particularities of each livestock operation [2].

Currently, computer programs are available that facilitate record-keeping and its analysis, drastically shortening the time needed for data processing [2,3]. These computer programs are part of the information and communication technologies (TIC) [4] and the management information systems (MIS) [5]. These programs are tools for the automatization of the existing processes, which will help to make changes easier to drive productivity growth [6,7,8].

The first studies to analyse the impact of such technology showed statistically significant associations between the use of computers for record-keeping and a higher production level [9,10], encouraging the agricultural sector to use MIS, as well as technical innovations [11]. One of the most important reasons for a low or zero adoption is the difficulty to quantify the direct effect of technology, as improvement is less tangible [5]. Van Asseldonk et al. [12] attributed this difficulty to the fact that the multidisciplinary processes on a dairy farm could be affected, for better or for worse, by the same decision. Moreover, most of the methods described in the literature focus on making a comparison before or after the adoption or on comparing users with non-users [13,14], without the existence of previously validated information [15].

Although these first approaches have been of great help, they may lead to an underestimation of the MIS effects, as it is difficult to determine at which point in time a producer starts to use these tools efficaciously [5]. Currently, there are databases whose integrity and consistency allow making a standardized comparison of the information. This will help the statistical models to be closer to reality, better showing the impact of the livestock information systems [16]. Such is the case of the veterinary automated management and production control program (VAMPP) Bovine, which has a centralized national database of 1758 users and a historical record-keeping from before 1990 only in Costa Rica, Central America. Furthermore, it is being used in almost 300 farms in Panama, Mexico, Colombia, Honduras, Guatemala, El Salvador, and the Dominican Republic.

The best way of spreading what is known as a new technology within the dairy sector is demonstrating that it offers important advantages related to the efficiency, efficacy, and income of a dairy farm [2]. Thus, the aim of this work is to evaluate the possible effects of an intensive use of the TIC and MIS on productivity levels and, particularly, the influence of the VAMPP Bovine implementation on dairy farms, helping to accredit the advantages that these technological tools can offer, under the assumption that everything that can be measured, can be improved.

## 2. Materials and Methods

The national specialized dairy herd in Costa Rica consists of 308,715 females located in 12,974 farms [17]. These farms are located at altitudes between 100 to 2500 m above sea level, in areas with an average temperature ranging between 18 and 30 °C and rainfall levels between 500 to 3500 mm per year. The most commonly used dairy breeds are Holstein and Jersey, with an average production of 22.1 and 16.8 kg of milk per day [18], respectively. Age at first calving for the same breeds are 29.9 and 28.6 months [18], whereas average days open amount to 143 and 122 days, respectively [18]. Feeding of dairy cattle is pasture-based, with varying degrees of supplementation [19]. From the total dry matter in the diet, the percentage contributed by grass varied from 18.8% to 71%, whereas percentage coming from concentrate ranged between 23% and 38% [19].

The current analysis was performed on information provided by the centralized national database administered by the Regional Information Technology Center for Sustainable Animal Production Centro Regional De Informatica Para La Produccion Animal Sostenible (CRIPAS) project [16]. Due to the historical origin of data, a non-experimental retrospective approach with a no-control time-series design [5] was used in the present analysis. The database contained information from 1758 specialized dairy farms with an average herd-size of 34 adult cows (minimum 5, maximum 660), all users of the VAMPP Bovine management information system. These farms had an average of 8.4 years (SD 7.5 years, minimum 0.25, and maximum 39) of accumulated information in the database. A subgroup of herds within this population was selected according to the following procedure.

### 2.1. Selection of Years per Herdand Variables

At herd level, personnel in charge upload the different kinds of events (productive, reproductive, and health-related) to the VAMPP system; this information is stored and sent periodically to the CRIPAS project [16] on a voluntary basis. The central database is dynamic; therefore, the herds can enter or exit the system at any time. Likewise, the recording and sending of information to the central database is voluntary, and thus not all herds have data updated for the last year, nor do they have data on every type of event.

For the present analysis, dairy herds with a minimum of five full consecutive years of information recorded in the VAMPP Bovine database were selected. Among the herds selected, the information used was restricted to a maximum of 10 years of follow-up, considering this a period long enough to appreciate the effects of the implementation of a new technology.

Regarding the importance for profitability in the dairy production systems, six performance variables were selected related to reproductive, productive, and health issues (Table 1). These variables were measured at the year per hered level as an average from all observations occurring within each calendar year and herd, and were defined as:

▪ Age at first calving (AFC): The average of the months since birth to the first calving for all the females with a first calving in the respective year per herd.
▪ Days open (DO): Average of the days since calving until the confirmed conception, for all the cows that conceived in the respective year per herd.
▪ Daily milk yield (DMY): Average in kilos per cow estimated from all the daily individual weighing in the respective year per herd.
▪ Productive life (PL): Average of years in production since the first calving until culling, for all the cows with record of cull in the respective year per herd.
▪ Incidence of mastitis during lactation (MAST): Percentage of lactations with at least one event reported with clinical mastitis, regarding the total of lactations started in the respective year per herd.
▪ Incidence of lameness during lactation (LAM): Percentage of lactations with at least one reported event of a lameness, regarding the total of lactations started in the respective year per herd.

#### Grouping of the Herds According to Their Adoption Level of the VAMPP^®^Bovine Program

Three adoption levels were defined according to the consistency and extent of the records in the previously defined variables (Table 1).

The low adoption level was formed by the herds that had at least 5 consecutive years of reproductive information only; the medium level included herds that had at least 5 consecutive years of reproductive as well as productive data; finally, the high adoption level included herds with at least 5 consecutive years of productive, reproductive, and health information.

### 2.2. Statistical Trend Analysis

The described variables were analyzed through a generalized linear mixed model using the GLIMMIX procedure [20] of the SAS program [21]. The statistical model was as follows:

*Y* = *β*_0_ + *β*_1_*ER* + *β*_2_*PB* + *β*_3_*HS* + *β*_4_*CP* + *β*_5_*AL_VAMPP* + *β*_6_*FY_VAMPP* + *β*_7_*AL_VAMPP* × *FY_VAMPP* + *eh* + *ih* + *ξ* (1)

where *Y* = response variables, measured at the herd per year level, as described in the previous section; *µ* = intercept; *ER* = fixed effect of the ecological region according to Holdridge [22] (humid forest (tropical, low mountain, or pre-mountain), very humid forest (tropical, low mountain, or pre-mountain), pre-mountain rainforest]; *PB* = fixed effect of the predominant breed (Holstein, Jersey, Holstein × Jersey crosses, crosses amongst dairy breeds, crosses amongst dairy breeds *× Bos indicus*, other breeds); *HS* = fixed effect of the herd size according to categories based on the number of cows in production in the respective year per herd (1 = between 5 and 20, 2 = between 20 and 40, 5 = 100 or more cows); *CP* = fixed effect of the calendar period, every 5 years (≤1990, 1991–1995, 1996–2000, 2001–2005, 2006–2010, ≥2010); *AL_VAMPP* = fixed effect of the VAMPP Bovine program adoption level (according to Table 1); *FY_VAMPP* = fixed effect of the follow-up year in VAMPP Bovine (1, 2, 3..., 10); *AL_VAMPP × FY_VAMPP* = fixed effect of the interaction between the adoption level and the follow-up year in VAMPP Bovine; *Eh =* random between-herds variation; *Ih =* random within-herd variation; *ξ* = random residual error.

CP effect was added to the model in order to account for possible fixed trends linked to different time periods. Examples of these are the evolution of the MIS during the time span considered. Farms may evolve in time, due to factors such as increased access to technologies in more recent years, better nutrition, genetics, or equipment. Weather or market conditions can also fluctuate over time.

FY_VAMPP effect was included in the model to measure the gradual impact of using the MIS, from year 1 (its first implementation in the herd) to year 10. CP and FY_VAMPP did not overlap, as the moment of first-time implementation of the MIS varied widely between herds.

*AL_VAMPP × FY_VAMPP* interaction effect was also added to the model, as the impact of MIS may be heterogeneous for herds with different adoption levels.

Random within-herd effect was added to the model to account for the interdependence that exists between the repeated measurements in successive years within the same herd, and this was modelled assuming a first order auto-correlation structure.

During the model construction, different probability distributions were assessed for each response variable. For the variables AFC, DO, DMY, and PL, the normal distribution provided a better adjustment, whereas for the variables MAST and LAM, a better adjustment of the lognormal distribution was obtained. According to the adjusted distributions, the minimum and maximum cut-off values were defined for each analyzed variable with the aim of reducing the bias caused by extreme values.

From the model solutions, marginal means were obtained for the different adoption levels and the follow-up years.

### 2.3. Cost–Benefit Analysis

To assess possible economic benefits derived from MIS implementation (VAMPP Bovine), an approach based on the partial budgeting analysis was used [23]. A base situation was assumed, with a herd of 100 cows in production, with a medium adoption level of the MIS, and a time horizon of 5 years of follow-up with the program. Moreover, it was assumed that efficiency levels were similar to the ones observed in the study herds of the medium adoption level.

The expected increase in costs (∆C) due to MIS implementation was estimated from initial investments in hardware and software, as well as the additional labor needed for collecting, entering, and analyzing the information in the system. On the side of income, the expected increase (∆I) was calculated on the basis of economic values for the traits DO and DMY. Economic values were defined as the expected change in gross margin (USD per cow per year) obtained as consequence of an improvement of 1 unit in the respective trait. These values were obtained from the stochastic simulation model developed by Vargas and Cuevas [24], modified for the current market conditions. To obtain an estimate of ∆I, economic values were multiplied by the expected rate of change in DO and DMY over the time horizon. This rate of change was assessed by fitting a linear regression on marginal means of DO and DMY during the 5 years of follow-up.

Gross margin (GM_MIS_ = ∆I − ∆C) and marginal return rate (MRR_MIS_ = ∆GM/∆C), attributed to the MIS implementation, were also calculated. Stochastic simulation was used to evaluate the sensitivity of the GM_MIS_ and MRR_MIS_ to marginal changes of ±10% in DO and DMY, as well as their economic value, herd size, and costs directly associated with MIS, such as hardware, software, and labor costs.

## 3. Results

After the application of the selection criteria, the final number of herds was 912, from which 389 (42.67%), 343 (37.6%), and 180 (19.7%) were classified as low, medium, and high adoption level herds, respectively. These herds ranged between a minimum of 5 and a maximum of 10 years of follow-up with the MIS, therefore, the number of herds per year classes varied extensively among the different traits under analysis (Table 2). The most complete information was in the reproductive performance domain variables, with a maximum of 7901 herds per year for the AFC trait. The variables with less information were related to the health domain, with 1287 herds per year for LAM.

### 3.1. Reproductive Performance Variables

From the analysis of the model, it was determined that the reproductive performance variables (AFC and DO) were significantly affected by almost all the evaluated effects, except for herd size (Table 3), which was not significant in the case of DO.

**Figure 1:**
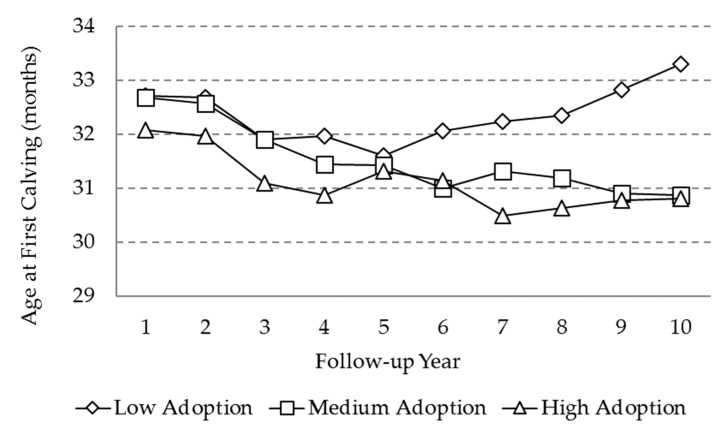
Marginal means for the variable “age at first calving” (AFC) as a function of adoption level and the follow-up year.

**Figure 2:**
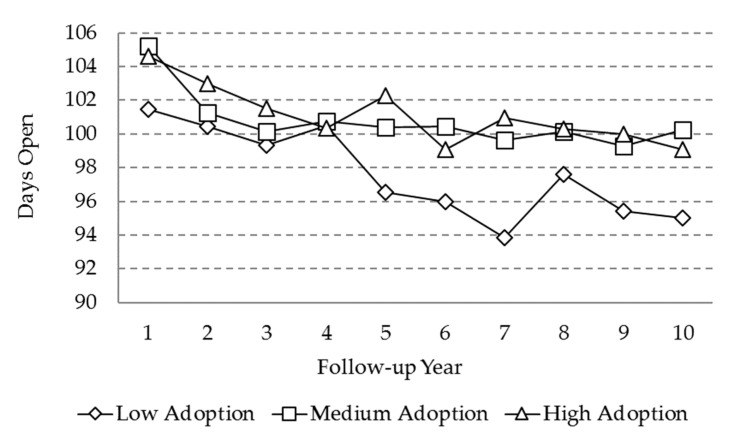
Marginal means for the variable “days open” (DO) as a function of adoption level and the follow-up year.

For both variables, there was a marked reduction in the first 4 follow-up years (Figure 1 and Figure 2). For the medium and high adoption level farms, the AFC showed a decreasing pattern until the fourth follow-up year in VAMPP Bovine, with a reduction of 2 months and 1 month, respectively, with fluctuations between the year 4 and 7. In the low level farms, an initial reduction of AFC during the first 5 years was observed, with a subsequent increase until year 10, finishing at almost 33.4 months, and a significantly higher (*p* < 0.001) value than the other two adoption level farms, which ended at nearly 30.9 months. The differences between medium and high level farms were not statistically significant through the follow-up period.

For the DO, a decreasing trend was observed in the three adoption level farms, with reductions of approximately 5 days in the high and medium level farms and 7 days for the low adoption level farms. The DO value for the latter one was significantly lower at the end of the period, as compared to the other two adoption level farms (Figure 2).

### 3.2. Production Variables

**Figure 3:**
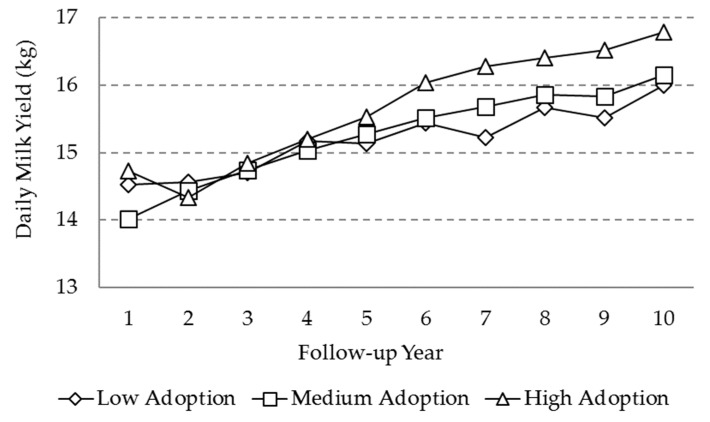
Marginal means for the “daily milk yield” (DMY) variable as a function of adoption level and follow-up year.

Daily milk yield (DMY) was significantly affected by breed, calendar period, agroecological zone, the follow-up year, and the random effects (Table 3), whereas the adoption level and its interaction with the follow-up year were not statistically significant. A statistically significant increasing trend in DMY was observed through the follow-up period (Figure 3), despite the adoption level. The rise in production was approximately 2 kg for the high adoption level farms and 1 kg for the medium and low adoption level farms. The increase was even more marked from year 3 onwards. Differences between the adoption level farms through the follow-up period were not significant (Table 3), but higher production was observed in the high adoption level farms toward the 10^th^ year.

**Figure 4:**
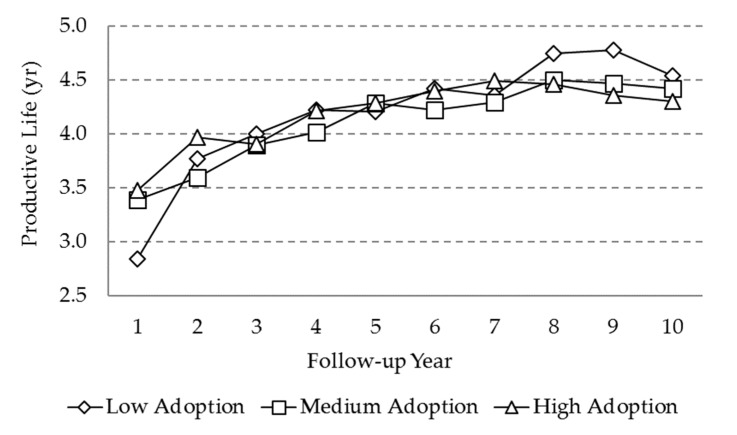
Marginal means for the ‘’productive life’’ variable as a function of adoption level and follow-up year.

Productive life (PL) was significantly affected by calendar period, the follow-up year, and random effects in the model (Table 3). A significant increasing trend for all three levels of adoption was also observed (Figure 4), with rises of approximately 1 year in PL, mainly from years 1 to 8. No significant differences were observed between the levels of adoption.

### 3.3. Health Variables

Both health variables (MAST, LAM) were significantly affected by the effects of herd size, calendar period, level of adoption, and the random effects (Table 3). The year of follow-up was only significant for MAST.

**Figure 5:**
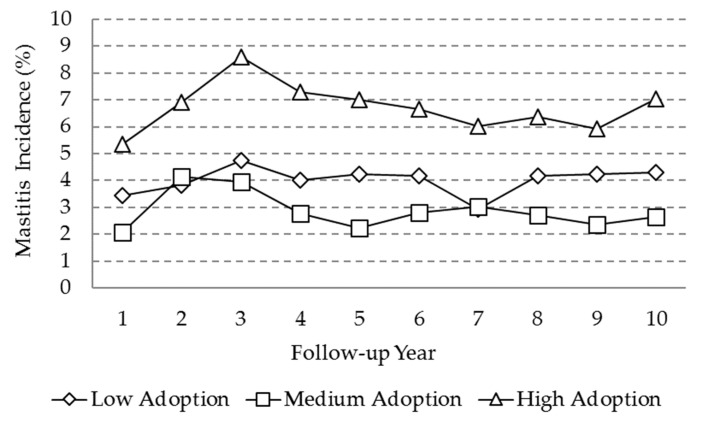
Marginal means for the ‘’incidence of mastitis’’ variable as a function of adoption level and follow-up year.

There was a higher incidence of mastitis for the high level of adoption over the 10 year period (Figure 5). The values fluctuated between 5% and 9% incidence for the high adoption level and between 2.0% and 5% incidence for the low and medium levels. These values correspond to geometric means, as they were obtained from exponentiation of logarithmic means. The trend throughout the follow-up period fluctuated in the three adoption level farms.

**Figure 6:**
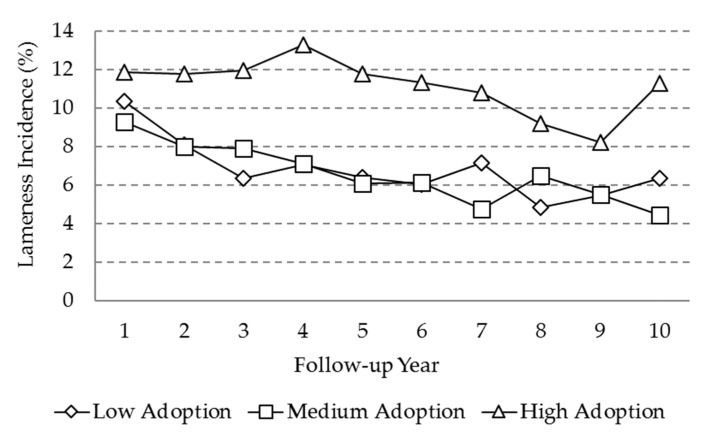
Marginal means for the “incidence of lameness” variable as a function of adoption level and follow-up year.

The incidence of lameness was also greater in the high adoption level farms (Figure 6), with values in the range between 8% and 13%. For low and medium adoption levels, a decreasing trend was observed through the 10 year period, starting at around 10% and ending at around 4%.

### 3.4. Cost–Benefit Analysis by Partial Budgetting

As can be observed in Table 4, partial budget analysis of MIS implementation for the base situation indicated an estimated gross margin of USD6890 with a marginal return rate of 163%.

From the sensitivity analysis, the variables that had more influence on gross margin (GM_MIS_) and marginal return rate (MRR_MIS_), were the expected annual change in the daily milk yield (DMY) and its economic value, both with a correlation of 0.58, and the size of the herd in production, with a correlation of 0.48 (Table 5).

A decrease of 10% in the expected annual change of the daily milk yield (DMY) resulted in a decrease of USD1001 in GM_MIS_ and 24% in MRR_MIS_. A reduction in herd size resulted in a reduction of USD829 in GM_MIS_ and 9% in MRR_MIS_. It is important to point out that in herds with less than 17 animals, the GM_MIS_ tended to reach negative values, as the increase in income was insufficient in covering the expenses of fixed costs of investment in hardware and software, leaving aside the costs of data collection and data entry.

The expected change in days open (DO) showed a smaller economic impact (correlation of −0.07) because the expected reduction in this trait was low. Other variables with similar impact were the costs per day for data entry and analysis (correlation of −0.11) and for data collection (correlation of −0.05). The variables related to the cost of hardware and software (correlation of −0.04) presented a lower impact because these are fixed costs at a medium or long term, becoming negligible as the size of the dairy operation increased.

## 4. Discussion

In the present study, a positive, non-experimental approach with a no-control time-series design [5] was used, on the basis of the empirical evidence provided by population data. Therefore, there was no control group as such, nor was it possible to have estimations of milk yield before the introduction of the program. The evaluation was based on the behavior of performance variables for users with different levels of adoption of the program, with a previous adjustment due to environmental, racial, or temporary effects, with a potential impact on the response variables. Although in these type of studies it is not possible to attribute—with certainty—direct causality, the availability of time series after the introduction of the MIS allowed for, in some cases, the observation of clear trends in the response variables, which may have been partially associated with the level of adoption and use of the MIS.

### 4.1. Reproductive Category

Average age of first calving (AFC) was lower in the medium and high adoption level herds as compared with the low adoption level. It is generally considered that the ideal average age at first calving is between 24 and 27 months for dairy cows in intensive systems [26]. For tropical regions, AFC averages are frequently close to 35 months [27]. These high values have been mainly associated with less favorable weather conditions, higher incidence of disease, pasture-based rearing systems, and the lack of high quality nutritional products in general [28].

In the current study, all AFC values were below 34 months. The trend to a decrease in the early years of follow-up suggests a contribution of the MIS to a better reproductive control of the herd, allowing animals to not remain “open” for long periods. A local study based only on recent data suggested that AFC had lowered down to 30.1 and 28.4 months for Holstein and Jersey cows, respectively [28], which means that AFC can also approach optimum values under tropical conditions.

For days open (DO), the observed average values in the present study remained below 105 days. The decreasing trend in the three adoption level herds suggests a positive impact of the information system. The restart of the ovarian activity post-calving was affected by race; age; the number of calvings; the pre-calving and post-calving nutritional conditions; and heat stress phenomena [29]. The ideal duration of the calving-to-conception interval ranges between 85 and 115 days to achieve the one calf a year goal commonly used in specialized dairy farms [28]. MIS are essential tools for the reproductive management of the herd because they provide action lists and allow for identification of problem cows in a timely manner.

The low adoption level herds presented significantly shorter DO compared to medium and high adoption level herds, even from the beginning of the follow-up period. One possible explanation for this result could be the greater abundance of crossbreeds Holstein × Jersey (DO = 96 days) cows in the group of low adoption level, coupled with a lesser abundance of the pure Holstein cows (DO = 104 days). Another possible explanation could be the antagonistic relationship between production and reproductive behavior [30], usually attributed to a negative energy balance after calving, as high levels of milk production demand more energy and nutrients [31]. As previously described, the high level of adoption tended towards an increased milk production. Epidemiological studies have also suggested that mastitis and lameness problems influence reproductive performance [32,33], and both variables (MAST and LAM) showed a higher incidence in the high adoption level herds.

### 4.2. Production Domain

Milk production is an important indicator, which reflects the health status, genetics, feeding management, handling, and the reproductive behavior of the animal [26]. Aviléz [34] found greater proximity between high milk production and the use of reproductive records, as well as a greater proximity between low to medium milk production levels and the absence of records. Similar cases, where records were integrated with the use of computers, agree with the improvement in milk production [9,10,35].

In this study, there was a significant increase in the daily milk production over the first 10 years of follow-up, regardless of the adoption level. This again suggests a positive impact of the use of the information system. Although the differences between adoption levels were not significant, the average milk production towards the end of the period was 1 kg more in the high adoption level herds as compared to the two other adoption levels herds. Milk production records of individual cows are essential for within-herd selection and to implement differential management in the herd [18]. MIS are helpful to identify superior cows, as well as those that should be discarded for low production.

Productive life also showed an increase during the first years of use of MIS, regardless of the adoption level. The culling and replacement of a dairy cow may happen anytime, due to involuntary reasons like disease, mortality, and infertility, or for voluntary reasons like an estimated low milk yield [36,37]. The ideal productive life of a dairy cow is based on several factors, such as the level of milk production by lactation, the value of a cull cow and the cost of the replacement [37]. A longer productive life may reduce replacement costs [38,39,40]. Values of productive life have been reported in Costa Rica as being 2.81 ± 0.17 years for breeds Bos Indicus × Brown Swiss and up to 4.26 ± 0.06 years for Holstein × Jersey [37]. Results of the present study suggest that MIS can help to prevent reproductive failure and involuntary culling thanks to better control and planning.

### 4.3. Health Category

The advantages of an early detection of health traits are closely linked with the costs of the treatment, replacement rates, the reduction of production losses, and the probability of culling [41]. In the case of dairy farms, mastitis is the most frequent health problem, and constitutes the main reason for the elimination of cows in the herds, with large economic impact for the dairy sector [42].

The incidence of clinical mastitis remained fluctuating throughout the follow-up period, and a significantly higher incidence in the high adoption level herds was observed from the first until the last year of the study. This result could be associated with several factors, such as the level of milk production, the size of the enterprise, or the quality level of management. The herds of a high level of adoption presented a trend to a higher milk production, which is a factor that may contribute to the event of mastitis [42]. On the other hand, the low adoption level herds had, on average, 15 animals less than high adoption level herds. It has been reported that in herds with a smaller number of animals, it is easier to detect the cases of mastitis, as well as to detect suspected or recurrent cases, which results in a decrease of the rate of mastitis at approximately 25% [12].

Regarding the incidence of lameness, a non-significant trend to a reduction in the herds of medium and low levels of adoption was observed. On the other hand, in herds of high adoption level, the incidence was higher throughout the follow-up period. This result could be related to factors such as housing facilities, the size of herd, or the level of milk production. The herds with a higher level of adoption of VAMPP Bovine are intensive specialized herds that correspond to semi-stalled and fully stalled systems, with a larger herd size [26,43,44]. The health of the hooves is severely affected when animals are in confinement with hard floors [45]. An increased incidence of lameness in cows of higher milk production, associated with high-energy diets, without a proper transition management, has also been reported [30,46].

A more intensive use of the MIS involves, per se, a greater control over events and records that are developed within each farm; it also involves an increased detection of health events and an accurate identification. For the variables of health, the fact that a lower incidence in low adoption herds was observed could also be associated with an under-registration of the information [42,44], as the variables of mastitis and foot lesions, as opposed to reproductive events, do not have a mandatory recording for the proper functioning of the VAMPP Bovine.

### 4.4. Cost–Benefit

Considering the investment in systems of information, economic criteria hardly arises because the profitability is generally unknown [5]. The marginal return rate for this study was 163% under certain conditions of herd size and milk production level, as a minimum number of animals are needed to justify the investment. In the literature available to the authors, the return on investment was in the range of 220% to 348% for systems of pig production and between 52% and 205% for dairy products [10]. This comparison suggests that the quantification not only depends on the costs of the technology represented in the software and hardware [35], but also in the type of production and in the years of information to be included in the studies.

Performing the economic estimation over a certain period, according to the annual behavior of variables, allowed a more detailed quantification. The benefits of technology do not occur as an immediate jump, they depend on the ability of the user obtained over the years to turn data into available and useful information, leading to an analysis, and adequate decision-making [12,47,48]. Yield is a result of the amount of a resource that is used, and specifying the value of the return on investment reduces the uncertainty of implementing new technologies in livestock production.

The present study also suggests low or no economic benefits for herds with less than 17 adult cows. This is important to notice, as the national average herd-size for dairy farms is 15 cows [17]. An alternative that has been implemented locally for these small farms is to join extension programs promoted by cooperatives, which significantly reduce the costs of implementing the MIS, while benefiting from the guidance and advice provided by these programs.

### 4.5. Final Considerations

Different factors are influential in convincing farmers to use decision support tools—most important among them are usability, cost-effectiveness, performance, relevance to user, and compatibility with compliance demands [49].

The VAMPP software is by far the MIS more frequently found in Costa Rican dairy farms. This is also reflected in the high average of follow-up years (8.4 years), a unique or at least very rare case in the Latin American countries. There are several reasons for this success. Commercial applications tend to focus on solving daily farm tasks and aim to generate income for the farmers through better resource management and field operations planning [50,51,52]. VAMPP differs from most of the similar commercial systems locally available, as it was created and distributed by a public academic entity, in which scientific and social development objectives prevail over economic interests.

One important finding of this study was that the low and medium adoption level farms are still the most common. The MIS is being used primarily for the recording of reproductive events, whereas the data for production and health events are reported less frequently. This finding reveals an under-utilization of the tool, which is generally attributed to the additional cost and management that represents measuring and recording this information, without any predictive certainty of the marginal benefit that it entails. Previous studies have also reported a very strong bias for adoption and use of MIS from the largest producers due to a higher management level [35].

In this sense, there is a need for the livestock sector for a better transfer of technology, which includes not only the adaptation and adoption of the tool but also a proper use of it. Furthermore, knowledge transfers to the farmers, regarding health events and production data of the herd, supportive for better management decisions, is still of paramount importance. Increased awareness of risks for reproduction and production diseases needs to be promoted. Milk production records of individual cows should be considered essential for within-herd selection and to implement differential management in the herd [18].

The interplay between the developers of MIS and end users should be favored by institutional actors such as universities and other organizations, such as dairy cooperatives, which could act as facilitators, providing training to farmers and feedback to developers [50,52]. For the VAMPP system, the continuous exchange of information between farmers, local cooperatives, and researchers has proven very beneficial, promoting the continuous improvement of the system and the collection of high-quality information at lower cost [5,16]. However, there is still much room for improvement on this issue.

## 5. Conclusions

Although it is difficult to assume that the behavior of the variables is completely due to the use of system of computerized records, the positive trend of the productive and reproductive performance variables suggests a strong influence of the MIS in the first years of follow-up. This is being reflected by livestock operations that are gradually learning to use information systems more effectively in order to carry out a more detailed observation of their production and to make more evidence-based decisions. The programs themselves have also evolved to have a better set of data evaluated more objectively. With these improvements, the analytical tools have made it possible to isolate and measure the real impacts of the MIS.

Finally, despite the years and multiple strategies to make recording systems a shared practice, the low levels of adoption are still the most common, revealing the need of the livestock sector for a better transfer of knowledge and technology, which includes not only the adaptation of the tool but also a proper use of it.

## Figures and Tables

**Table 1:** Adoption levels according to consistency and extent of record keeping in the veterinary automated management program for the production control (VAMPP) Bovine system on the key variables in the reproductive, productive, and health areas.

Parameters	Score	Adoption Level ^A^		
		Low	Medium	High
Reproductive category				
Age at first calving (AFC)	Months	(+)	(+)	(+)
Days open (DO)	Days	(+)	(+)	(+)
Productive category				
Daily milk yield (DMY)	kg	(-)	(+)	(+)
Productive life (PL)	Years	(-)	(+)	(+)
Health category				
Mastitis incidence (MAST)	%	(-)	(-)	(+)
Lameness incidence (LAM)	%	(-)	(-)	(+)

^A^ (+) Availability or (-) non-availability of at least 5 consecutive years of recorded information in VAMPP Bovine for the respective variable.

**Table 2:** Number of years per herd classes, average ($\bar{x}$), standard deviation (SD), and 95% confidence limits for the variables age at first calving (AFC), days open (DO), daily milk yield (DMY), productive life (PL), mastitis incidence (MAST), and lameness incidence (LAM).

Variable	Score	Herds per Year	$\bar{x}$	SD	95% Confidence Limits	
					Lower	Higher
Age at first calving (AFC)	Month	7901	31.3	4.8	31.2	31.5
Days open (DO)	Day	7857	100.6	16.1	100.2	100.9
Daily milk yield (DMY)	kg	4363	16.7	4.5	16.6	16.9
Productive life (PL)	Year	6326	4.02	1.45	3.98	4.06
Mastitis incidence (MAST)	%	1675	10.9	10.7	10.4	11.5
Lameness incidence (LAM)	%	1287	17.9	17.4	16.9	18.9

**Table 3:** Significance values (P) of fixed and random effects over the variables of age at first calving (AFC), days open (DO), daily milk yield (DMY), productive life (PL), mastitis incidence (MAST), and lameness incidence (LAM).

Effects of the Model	Significance Values (*p*)					
	AFC	DO	DMY	PL	MAST	LAM
Fixed						
Herd size	<0.001	<0.97	0.26	0.48	<0.001	<0.001
Predominant breed	<0.001	<0.001	<0.001	0.44	0.30	0.58
Agroecological zone	<0.001	<0.01	<0.001	0.65	0.71	0.80
Calendar period	<0.001	<0.001	<0.01	<0.001	<0.01	<0.001
Adoption level VAMPP	<0.001	<0.001	0.35	<0.48	<0.001	<0.001
Follow-up year VAMPP	<0.001	<0.001	<0.001	<0.001	<0.01	0.09
Adoption level × follow-up year	<0.001	<0.001	0.15	<0.01	0.60	0.63
Random ^A^						
Between herds	<0.001	<0.001	<0.001	<0.001	<0.001	<0.001
Intra herds	<0.001	<0.001	<0.001	<0.001	<0.001	<0.001

^A^ For the random effects, the reported *p*-values were obtained from the Z Wald test.

**Table 4:** Economic analysis by partial budget applied to a base situation assuming a dairy herd with 100 adult cows, with a medium adoption level of the MIS (VAMPP Bovine) over a time horizon of 5 years.

Parameters	Scale		Value
Expected increase in costs:			
Investment in hardware ^A^	USD/year		$140
Investment in software ^B^	USD/year		$140
Data collection ^C^	USD/year/100 cows		$188
Data entry and information analysis ^C^	USD/year/100 cows		$376
Total increase of costs (∆C)	USD/5 year/100 cows		$4219
Expected increase in income:		DO (d)	DMY (kg)
Expected annual change in trait ^D^	unit/cow/year	−1.02	0.31
Economic value of the trait ^E^	USD/unit/cow/year	−$2.15	$64.6
Increase in income by trait	USD/5 year/100 cows	$1097	$10,013
Total increase of income (∆I)	USD/5 year/100 cows		$11,110
Gross margin (∆GM_MIS_ = ∆I − ∆C)	USD/5year/100 cows		$6890
Marginal return rate (MRR_MIS_ = ∆GM/∆C)	%		163.3%

^A^ Hardware: cost of desktop computer + printer: USD700, distributed over a useful life of 5 years. ^B^ Software: cost of the license of the VAMPP Bovine program with a capacity for handling three herds: USD700, with updates every 5 years. ^C^ Assuming 7.6 (data collection) + 15.2 (data entry and analysis) annual additional days with a cost of $24.7 per day based on minimum wages of the Ministry of Labor and Social Security of Costa Rica [25]. ^D^ Annual change in trait: estimated from the slope obtained by linear regression of the marginal means of days open (DO, Figure 2) and daily milk yield (DMY, Figure 3) over the first 5 years of follow-up with VAMPP Bovine in herds with a medium level of adoption of the MIS. ^E^ Economic value of the trait was defined as the expected change in gross margin (USD per cow per year) as a result of an improvement of one unit for the respective trait. These values were obtained from a modified version of the bio-economic model of simulation of Vargas and Cuevas [24], updated with prices of supplies and products corresponding to March 2017.

**Table 5:** Impact of variables of milk yield, days open, herd-size, and costs on gross margin (GM_MIS_) and the marginal return rate (MRR_MIS_) for the use of the MIS.

Variables	Correlation ^A^ (r)	GM_MIS_ ($)	Change ^B^ ($)	MRR_MIS_ (%)	Change ^B^ (%)
**Base situation:**		(6890)		(163.3)	
Annual change in daily milk yield	0.58				
−10% (0.28 kg/year)		5889	−1001	139.6	−23.7
+10% (0.34 kg/year)		7892	1002	187.0	23.7
Annual change in days open	−0.07				
−10% (−1.12 d)		7000	110	165.9	2.6
+10% (−0.92 d)		6781	−109	160.7	−2.6
Herd size	0.48				
−10% (90 cows)		6061	−829	153.9	−9.4
+10% (110 cows)		7719	829	171.5	8.2
Costs (data entry and analysis)	−0.11				
−10% ($22.2)		7078	188	175.6	12.3
+10% ($27.2)		6703	−187	152.1	−11.2
Cost (data collection)	−0.05				
−10% ($22.2)		6984	94	169.3	6.0
+10% ($27.2)		6796	−94	157.6	−5.7
Cost (software and hardware)	−0.04				
−10% ($630)		6960	70	167.8	4.5
+10% ($770)		6820	−70	159.0	−4.3

^A^ Correlation between the respective row variable and the output variables (gross margin and marginal return rate) obtained from the simulation procedure. ^B^ Change in gross margin or marginal return rate compared to the base situation.
